# Biochemical and Structural Properties of a High-Temperature-Active Laccase from *Bacillus pumilus* and Its Application in the Decolorization of Food Dyes

**DOI:** 10.3390/foods11101387

**Published:** 2022-05-11

**Authors:** Tao Li, Xiuxiu Chu, Zhaoting Yuan, Zhiming Yao, Jingwen Li, Fuping Lu, Yihan Liu

**Affiliations:** 1 Key Laboratory of Industrial Fermentation Microbiology, Ministry of Education, Tianjin Key Laboratory of Industrial Microbiology, College of Biotechnology, Tianjin University of Science and Technology, Tianjin 300457, China; 201096@xxmu.edu.cn (T.L.); chuxiuxiu@126.com (X.C.); yuanzhaoting@mail.tust.edu.cn (Z.Y.); yaozhiming611@126.com (Z.Y.); lijingwen619@126.com (J.L.); 2 School of Life Science and Technology, Xinxiang Medical University, Xinxiang 453003, China; 3 State Key Laboratory of Food Nutrition and Safety, Tianjin 300457, China

**Keywords:** *Bacillus pumilus*, molecular dynamic simulations, enzymatic characterization, food dye decolorization

## Abstract

A novel laccase gene isolated from *Bacillus pumilus* TCCC 11568 was expressed, and the recombinant laccase (rLAC) displayed maximal activity at 80 °C and at pH 6.0 against ABTS. rLAC maintained its structural integrity at a high temperature (355 K) compared to its tertiary structure at a low temperature (325 K), except for some minor adjustments of certain loops. However, those adjustments were presumed to be responsible for the formation of a more open access aisle that facilitated the binding of ABTS in the active site, resulting in a shorter distance between the catalytic residue and the elevated binding energy. Additionally, rLAC showed good thermostability (≤70 °C) and pH stability over a wide range (3.0–10.0), and displayed high efficiency in decolorizing azo dyes that are applicable to the food industry. This work will improve our knowledge on the relationship of structure–function for thermophilic laccase, and provide a candidate for dye effluent treatment in the food industry.

## 1. Introduction

Laccases (EC 1.10.3.2), as multi-copper enzymes can catalyze the mono-electronic oxidation of substrates (aromatic amines and phenolic compounds) to yield water as a by-product with molecular oxygen as an electron acceptor [1]. Thus, laccases are more eco-friendly and potentially superior to other oxidoreductases, which require expensive cofactors (NAD^+^ or NADP^+^) or the harmful hydrogen peroxide to complete the oxidative reaction [2,3]. Additionally, the substrate scope of laccases can be expanded to polyphenol polymers and other substrates with the assistance of redox mediators, such as syringaldazine, 2,6-dimethoxyphenol (2,6-DMP), and 2,2′-azino-bis-(3-ethylbenzothiazoline-6-sulphonic acid) (ABTS) [4,5]. Because of their capacity for oxidizing numerous substrates (such as methoxy-substituted monophenols, diphenols, aliphatic, and aromatic amine), laccases demonstrate a huge potential for numerous biotechnological processes, including food industry wastewater treatment, pulp bio-bleaching, and xenobiotics bioremediation [6].

Laccases can be acquired from different sources, such as bacteria, fungi, actinomycetes, and plants. Fungal laccases show more favorable characteristics for commercial applications in comparison to other laccases from plants and bacteria, because of their high reduction potential [7]. However, fungal laccases also demonstrate some drawbacks, including low fungal growth rate and being operative only under low temperature and pH conditions. They are often deactivated in some industrial sections, such as food and textile effluent decolorization, under the extreme conditions that occur, including high temperature, extreme pH value, or high ionic strength [8].

Thermophilic enzymes are favorable for use in both fundamental research and industrial applications [9]. A lower risk of microbial contamination and higher reaction rates were obtained when using thermophilic enzymes at high temperatures [10]. Moreover, thermophilic enzymes are favorable for some application scenarios that feature high temperatures, such as the treatment of dye effluent that is discharged during food processing. Therefore, over the past decade, more and more attention has been paid toward laccases with bacterial origins that show an excellent degree of catalytic ability at high temperatures, such as *Bacillus*, *Staphylococcus*, *Streptomyces*, *Geobacillus*, *Aquisalibacillus*, *Rhodococcus*, *Lysinibacillus*, *Pseudomonas*, *Proteobacterium*, *Enterobacter*, *Delfia*, and *Alteromonas* [11].

The most well-known bacterial laccase to date is CotA from *Bacillus subtilis*, which is a constituent of the endospore coat. It demonstrates a strikingly intrinsic thermostability with an optimum temperature of 75 °C, as well as a half-life of 2 h or so at 80 °C [12]. Recently, more and more laccases of several *Bacillus* species, including *B. licheniformis*, *B. amyloliquefaciens*, *B. halodurans*, *B. subtilis*, *B. thuringiensis*, and *B. vallismortis* have exhibited excellent rates of catalytic performance over a broad pH range and at high temperatures [13]. Referring to the heat-resistance features of laccase, researchers have reported their experiment evidence and presented a different proposal. For instance, salt bridges and glycosylation played a vital role in maintaining the structural integrity of thermophilic laccase from *Trametes versicolor* [14]. A similar stimulative effect on laccase activity via thermal incubation has also been demonstrated for some fungal laccases derived from *Physisporinus rivulosus*, *Marasmius quercophilus*, *Melanocarpus albomyces*, and *Fomes sclerodermeus* [15,16]. Nevertheless, studies for interpreting the thermo-stable mechanism of *Bacillus* laccase at the molecular level are still rare. Therefore, more research is still required to elucidate the structure–function relationship of bacterial laccases at high temperature. According to the peculiar characteristics of bacterial laccases, including a high tolerance for temperature, an alkaline environment, and salt, it is still necessary to discover novel bacterial laccases to enlarge our knowledge on the bacterial laccases and to expand the range of obtainable biocatalysts for different industrial processes performed under harsh conditions. Here, a novel laccase coding sequence was amplified from *B. pumilus* TCCC 11568 and heterologously expressed to identify its catalytic and biochemical properties. Molecular dynamics (MD) simulations were employed to explore the molecular mechanisms for the thermophilic features of this laccase. The ability of this novel laccase to decolorize synthetic dyes at high temperatures was then investigated.

## 2. Materials and Methods

### 2.1. Strains, Plasmid, and Chemicals

*E. coli* JM109, *E. coli* BL21 (DE3), and plasmid pET-22b (+) were deposited in our lab. Pyrobest DNA Polymerase, restriction enzymes, pMD18-T vector cloning Kit, Plasmid Mini Kit, T4 DNA ligase, Gel Extraction and Purification Kit, and DNA Extraction Kit were supplied by TAKARA Bio Inc. (Dalian, China). ABTS and synthetic dyes (azophloxine, etythrosine, Sunset Yellow, Ponceau 4R, Amaranth, Indigo Carmine, Acid Orange II, Congo Red) were ordered from Sigma-Aldrich (St. Louis, MO, USA).

### 2.2. Strain Screening and Cultivation

The forest soil sample was collected in Guangzhou, Guangdong Province, China. The screening procedure was performed according to the reported method for isolating *Klebsiella pneumonia*, which produced a new pH-stable laccase [17]. Briefly, 10 g of soil was transferred to a sterile saline solution (0.9% NaCl, 100 mL). Subsequently, 1 mL of the suspension was dispersed in 5 mL of Luria-Bertani (LB) medium (tryptone 10 g/L, yeast extract 5 g/L, and NaCl 10 g/L) with shaking for 30 min at 200 rpm and 37 °C. The enriched cells were spread on LB plates supplemented with 0.2 mM Cu^2+^ (copper sulfate) and incubated at 37 °C for 24 h. Several drops of syringaldazine (SGZ)/absolute ethanol (0.1%, *w*/*v*) were added to the bacterial colonies to distinguish the laccase-producing strains by their pink color. Then the obtained colonies that showed a pink color were picked up and purified using single-colony separation.

### 2.3. Phylogenetic Analysis of the Laccase-Producing Strain

The 16S rDNA was amplified with the genomic DNA of the laccase-producing strain according to the method described previously [17]. The homologous sequences of the resulting 16S rDNA sequence were searched with GenBank BLAST (http://www.ncbi.nlm.nih.gov/BLAST/ accessed on 12 March 2020). Subsequently, a bootstrap consensus tree was built using MEGA 6.0 software with the neighbor-joining method for phylogenetic analysis [18]. To further identify this strain, taxonomic analysis was conducted as noted in Bergey’s Manual of Determinative Bacteriology.

### 2.4. Heterologous Expression of Laccase

The gene encoding laccase (*lac*) was amplified with two primers, Lac-F (5′-CGCGGATCCGATGGCACTGGAAAAATTTG-3′; the underlined bases encode the *Bam*HI site) and Lac-R (5′-ACGCGTCGACCTGCTTATCCGTGACGTCC-3′; the underlined bases encode the *Sal*I site) from the genomic DNA of *B. pumilus*. After double digestion with *Bam*HI and *Sal*I, it was integrated into the *Bam*HI-*Sal*I-linearized expression plasmid pET-22b (+) to construct the plasmid pET-*lac*. It was then transformed into *E. coli* BL21 (DE3). A positive colony was initially selected to grow at 37 °C in LB medium containing ampicillin (100 μg/mL) for 12 h. Subsequently, the preculture (1 mL) was diluted with 50 mL of fresh LB medium with ampicillin (100 μg/mL). When OD_600_ reached 0.6–0.8, 1 mM isopropyl-β-D-1-thiogalactopyranoside (IPTG) was used to induce the recombinant laccase (rLAC) at 16 °C for 20 h. *E. coli* BL21 cells harboring the empty plasmid pET-22b (+) were used as the control.

### 2.5. Molecular Docking and Molecular Dynamics Simulation Analysis of rLAC

The software AutoDock Vina was used to perform molecular docking between the rLAC and the ligand. The 3D structure of rLAC was BLAST-searched in the UniProt database using SWISS-model (https://swissmodel.expasy.org/ accessed on 15 May 2021). A protein structure (Protein Data Bank entry 1GSK) with more than 70% homology was used as the template for homology modeling. The 3D structure of ABTS was obtained from PubChem (https://pubchem.ncbi.nlm.nih.gov/ accessed on 15 May 2021). A method of semi-flexible docking was utilized to allow the chemical bonds of the ligand to freely rotate, while fixing the coordinates of the atoms in rLAC. The grid parameters were set to build a sphere grid near the active sites of rLAC1 (H419, C492, H497, and M502) with a radius of 5.0 Å in the docking area.

The docking conformation with the lowest protein–ligand binding energy was selected for molecular dynamics simulation by Gromacs 5.1.4, combined with the AMBER99SB forcefield. The parameters for the molecular dynamics simulations were set based on the previous study [19,20], in which the SPC216 model was set as the water molecule. The simulation system was neutralized by a total of 11 Na^+^, which were added into a cubic box with a distance of 1.5 nm in all three dimensions. The incorrect geometries and collisions among the atoms in the simulation system were removed by the steepest descent minimization method, using a maximum number of 50,000 steps. Equilibration simulations for the solvent around the protein were performed under constant volume–temperature (NVT) and constant pressure–temperature (NPT) ensembles under harmonic restraints. Short-range van der Waals interactions and short-range electrostatic interactions were truncated smoothly at 1 nm. The leap-frog integrator algorithm with a step size of 2 fs was utilized to integrate the equation of motion for equilibrium dynamics. The LINCS algorithm was used to constrain the hydrogen bonds. The Particle Mesh Ewald (PME) method was used to calculate the long-range electrostatic interactions with a grid size of 0.16 Å. The pressure (1 bar) and temperature (300 K) under the isothermal–isostatic (NPT) ensemble were maintained using Parrinello-Rahman and the V-rescale method, respectively. Both the NVT and NPT simulation times were set as 100 ps and the final MD simulations were carried out for 100 ns. The root-mean-square fluctuation (RMSF) and the root-mean-square deviation (RMSD) were analyzed using the *gmx rmsf* and *gmx rms* commands respectively. The snapshots were performed using Visual Molecular Dynamics (VMD) 1.9.4.

The approach of MM/PBSA (Molecular Mechanics Poisson Boltzmann Surface Area) was utilized for energy decomposition according to the trajectory of the molecular dynamics simulations. The binding energy within a time period of 0~10 ns was calculated using the tool g_mmpbsa.py and the software Numpy (Numerical Python). The binding free energy is calculated using Equation (1):

(1) $$\Delta G_{\mathrm{bind}}=\Delta E_{\mathrm{vdw}}+\Delta E_{\mathrm{ele}}+\Delta G_{\mathrm{solv}}+\Delta G_{\mathrm{SASA}}$$

Among them, Δ*G*_bind_ stands for the binding free-energy, Δ*E*_ele_ stands for the electrostatic interaction, Δ*E*_vdw_ stands for the van der Waals force, Δ*G*_SASA_ stands for the non-polar contribution energy calculated using the solvent accessible surface (SASA), and Δ*G*_solv_ stands for the solvent free-energy.

### 2.6. Purification of rLAC

Cells expressing rLAC were centrifugally harvested via centrifugation at 8000× *g* and 4 °C for 15 min, and then resuspended in 20 mM Tris–HCl buffer (pH 7.0) with 500 mM NaCl and 20 mM imidazole. Subsequently, the cells were disrupted via sonication at 320 W with 4 s strokes and 3 s intervals. After centrifugation (12,000 × *g*, 30 min), the supernatant was injected into a nickel-nitrilotriacetic acid (Ni-NTA) agarose gel column (Shenggong, Shanghai, China). rLAC was eluted using Tris-buffer containing 500 mM imidazole and 500 mM NaCl (pH 7.0) after removing the impurities. After dialysis, the purity and molecular mass of rLAC were determined using sodium dodecyl sulfate polyacrylamide gel electrophoresis (SDS-PAGE) analysis.

### 2.7. Enzyme Assay

The activity of rLAC was determined with ABTS as the substrate at 80 °C using the previously described method [21,22]. Concisely, the enhanced absorbance at 420 nm (ε_420_ = 36,000 M^−1^ cm^−1^) was monitored for ABTS (6 mM) oxidation in 0.1 M citrate–phosphate buffer (pH 6.0). One unit of the activity for laccase was designated as the amount of laccase that oxidized 1 µmol of substrate per minute.

### 2.8. Characterization of rLAC

The optimal temperature of rLAC was determined with ABTS as the substrate at different temperatures from 30 °C to 90 °C. To identify the optimal pH of rLAC, the enzymatic reaction was conducted over a pH range of 3.0 to 9.0 with citrate–phosphate buffer (0.1 M, pH 3.0 to 8.0) or glycine–sodium hydroxide buffer (0.1 M, pH 9.0). The relative enzymatic activity was determined with the maximal activity as 100%.

To identify the thermostability of rLAC, it was incubated at three temperatures (60 °C, 70 °C, and 80 °C) for 0 h to 2 h without substrate. The pH stability was identified by keeping the purified rLAC at pH 3.0, 7.0, and 10.0 (4 °C) for various periods (0–10 days). After incubation, the residual activities of the samples were measured with the substrate ABTS under optimal conditions (80 °C, pH 6.0). The activity of the initial rLAC was taken as 100%.

The effects of the metal ions (K^+^, Na^+^, Cu^2+^, Ca^2+^, Fe^2+^, Fe^3+^, Zn^2+^, Mn^2+^, Co^2+^, Mg^2+^, and Ba^2+^), as well as the inhibitors (EDTA, SDS, L-cysteine, dithiothreitol, and β-mercaptoethanol) on rLAC activity, were assayed with ABTS. rLAC and metal ions or inhibitors were mixed before adding ABTS to determine the relative activity. The laccase activity measured without adding metal ion or inhibitor was marked as 100%.

### 2.9. Dye Decolorization

Decolorization was conducted using azo dyes, such as etythrosine (λ_max_ = 520 nm), azophloxine (λ_max_ = 530 nm), Sunset Yellow (λ_max_ = 480 nm), Ponceau 4R (λmax = 510 nm), Indigo Carmine (λ_max_ = 612 nm), Amaranth (λ_max_ = 520 nm), Acid Orange II (λ_max_ = 480 nm), and Congo Red (λ_max_ = 500 nm). Decolorization was assayed with or without the mediator (acetosyringone, syringaldehyde, and ABTS). The reaction solution (6 mL) for decolorization was composed of individual dye (azophloxine, Indigo Carmine, Ponceau 4R, 50 mg/L; Amaranth, Acid Orange II, 100 mg/L; etythrosine, Congo Red, Sunset Yellow, 200 mg/L), purified rLAC (80 U), 0.1 M buffer with different pH (citrate–phosphate buffer, pH 5.0 and 7.0; 0.1 M glycine–sodium hydroxide buffer, pH 9.0), and 0.1 mM mediator. After a 6 h reaction at 60 °C, the decolorization ability of each dye was identified by recording the reduction of each dye at the maximum absorbance using the equation: decolorization (%) = [((initial absorbance) − (final absorbance))/(initial absorbance)] × 100%, reflecting the reduction in concentration because of the oxidation reaction by rLAC. The control reactions were carried out with no laccase under the same conditions.

## 3. Results and Discussion

### 3.1. Identification of the Strain with Laccase Activity

Numerous studies have been focused upon bacterial laccases over the past decade, especially for those derived from the *Bacillus* genus, due to their advantages over fungal laccases, such as a higher pH stability, wider pH adaptation, and thermostability [12,23]. In this work, a bacterial strain demonstrated a high degree of laccase activity towards syringaldazine among 216 bacteria screened from a soil sample gathered from Guangdong Province of China. After aligning the 16S rDNA sequence of this strain with BLAST included in the NCBI database, the result suggested this strain was a member of the *Bacillus* family. Phylogenetic tree analysis suggested that this strain was most closely associated with *B. pumilus* (Figure S1 in Supplementary Materials).

The morphological properties of this strain are as follows: rod-shaped, Gram-positive, rough-surfaced, and with spore-producing colonies (data not shown). Therefore, it was finally characterized and named *B. pumilus* TCCC 11568 on the basis of its biochemical tests and morphological characteristics, along with the 16S rDNA analysis. In summary, we isolated a new *Bacillus* strain showing laccase activity from a soil sample, and our study enriched the family of laccase-producing bacteria.

### 3.2. Heterologous Expression of Laccase

The obtained laccase gene (*lac*) (GenBank: MT150577) contains 1530 bp nucleotides coding for 510 amino acids (calculated molecular mass: 57 kDa). Multiple protein sequence alignments indicated that its four histidine-rich regions for copper-binding were greatly conserved with other laccases. A high amino acid identity was demonstrated with the following laccases of other *Bacillus* species, such as 97.65% to *B. pumilus* ATCC 7061 [24], 94.71% to *B. pumilus* W3 [25], 67.96% to *B. vallismortis* fmb103 [26], 66.54% to *B. amyloliquefaciens* TCCC 111018 [27], 66.21% to *B. subtilis* X1 [28], 64.98% to *B. velezensis* TCCC 111904 [29], and 61.36% to *B. licheniformis* ATCC 14580 (Figure S2) [30].

**Figure 1:**
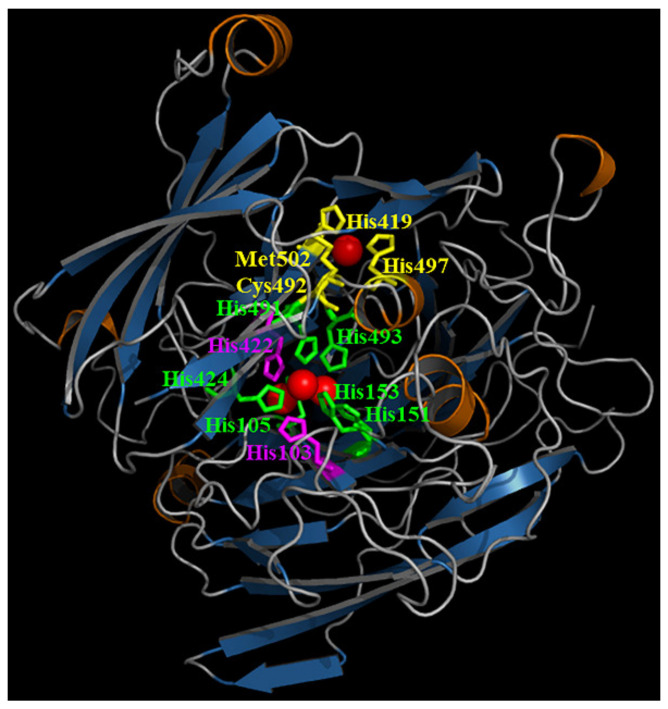
Homology model of the *B. pumilus* TCCC 11568 laccase. Three copper centers, including Type 1, Type 2, and Type 3, are depicted in yellow, purple, and green, respectively. The α-helices and β-sheets are denoted in orange and blue, respectively. Cu^2+^ located at the active site of rLAC is labeled in red.

The tertiary structure of the laccase from *B. pumilus* TCCC 11568 was built using homology modeling using the crystal structure (PDB ID: 2WSD) of laccase from *B. subtilis* MB24 as the template (Figure 1).

*Bacillus* laccases commonly have a highly conserved catalytic site with four copper atoms bound to the T1, T2, and T3 copper centers [31]. Similarly, three Cu oxidase domains, such as T1 (H419, C492, H497, and M502), T2 (H103 and H422), and T3 (H105, H151, and H493/H153, H424, and H491), also exist in the active center of the *B. pumilus* TCCC 11568 laccase.

In this work, a significant band of approximately 57 kDa was observed on the SDS-PAGE gel of the cell extracts, while no band was found in the cell extract of the control (Figure S3a). A target band with a molecular mass of approximately 57 kDa was detected on the SDS-PAGE gel after purification (Figure S3b). The obtained molecular mass was similar to some other reported bacterial laccases, including *B. amyloliquefaciens* [27] and *B. halodurans* C-125 (56 kDa) [32]. Its specific activity towards ABTS was 190 U/mg, which was notably higher than other *Bacillus* laccases to ABTS, such as *B. amyloliquefaciens* LC02 (20.7 ± 1.2 U/mg for ABTS) [33]. According to the sequence alignment, protein modelling, and enzyme activity analysis, it can be concluded that a new laccase with a high level of activity and a conserved catalytic motif that has been observed in other reported laccases was obtained in our study.

### 3.3. Effect of Temperature and pH on the Activity and Stability of rLAC

**Figure 2:**
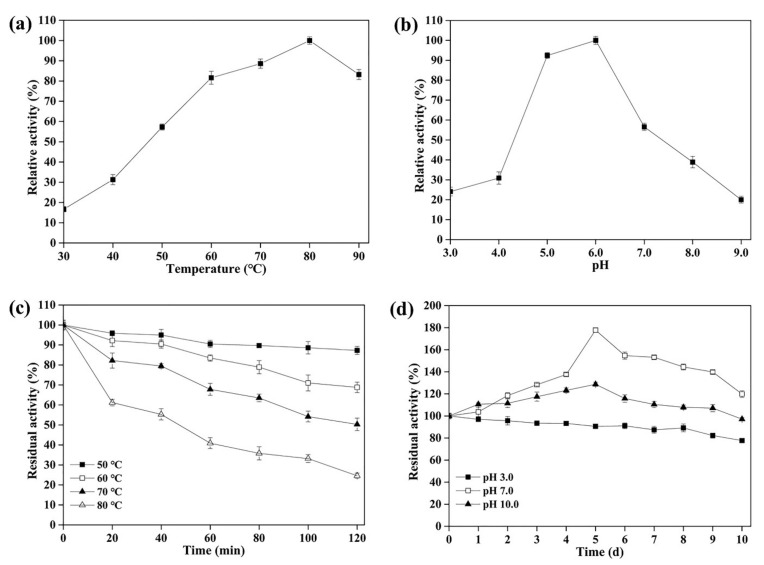
Effects of temperature or pH on the purified rLAC. (**a**) The laccase activity was recorded at different temperatures (30–90 °C) and (**b**) pH values. (**c**) The thermostability of rLAC was evaluated through monitoring the residual activity after incubation at 50 °C, 60 °C, 70 °C, and 80 °C for different lengths of time (0–120 min). (**d**) The pH stability was investigated through measurements of its residual activity after incubation for different periods (0–10 d) at 4 °C and pH 3.0, 7.0, and 10.0. All assays were conducted in triplicate, and the data were exhibited as mean ± SD.

The maximum activity of rLAC for ABTS oxidation was detected at 80 °C, and a relatively high activity level was sustained at 60–90 °C (Figure 2a). To investigate its thermostability, the residual activities were determined after incubation at differing temperatures (50–80 °C) for 0 min to 120 min (Figure 2c). It suggested that rLAC was quite stable at 50 °C or 60 °C, and retained 87.3% or 70% of the original activity at these temperatures after a 120-min incubation, respectively. Although rLAC showed an evidently decreased stability after incubation at a high temperature (70 °C or 80 °C), it still retained 50.3% and 24.6% of the original activity after a 120-min incubation, respectively (Figure 2c).

The high optimum temperature was one of the most significant properties demonstrated by the bacterial laccases, especially for those derived from the *Bacillus* genus, such as 80 °C for CotA laccase from *B. amyloliquefaciens* TCCC 111018 [27], *B. velezensis* [29], and *B. licheniformis* [34]. By contrast, a similar phenomenon was not observed for fungal laccases whose optimum temperatures were usually below 60 °C. The optimal temperatures for laccases from *Cerrena unicolorstrain* GSM-01, *Trametes* sp. F1635, and *Aureobasidium melanogenum* strain 11-1 were 45 °C, 50 °C, and 40 °C, respectively [35,36,37]. Compared to fungal laccases, the high thermostability is another impressive feature of bacterial laccases. The thermostability of rLAC from *B. pumilus* TCCC 11568 was similar to laccases isolated from other *Bacillus* strains, such as *B. amyloliquefaciens* TCCC 111018 [27], and *B. tequilensis* SN4 [38]. Moreover, its thermostability was even higher than *B. licheniformis* DSM 13 laccase, which was deprived of approximately 92% of its initial activity after 1 h of incubation at 80 °C [33]. Thus, rLAC might be directly applied in treating hot effluents from the dyeing process, which is generally performed under high-temperature conditions [39]. In addition, using thermostable laccase to treat hot textile effluents is economically attractive because the recycling of hot water saves a significant amount of energy in the process.

rLAC was capable of oxidizing ABTS over a wide pH range and demonstrated its maximum activity at pH 6.0 (Figure 2b), which was higher than most *Bacillus* laccases, such as *B. velezensis* TCCC 111904 [29], *B. amyloliquefaciens* TCCC 111018 [27], *B. vallismortis* fmb-103 [26], *B. subtilis* X1 [28], and *B. clausii* KSM-K16 [13]. Generally, fungal laccases are merely stable under acidic and neutral pH conditions, but bacterial laccases are stable under alkaline conditions [40]. Additionally, rLAC exhibited a higher degree of stability (Figure 2d) than other *Bacillus* laccases over a wide pH range, such as the *B. velezensis* TCCC 111904 laccase, which retained 67.6% of its initial activity after incubation at 4 °C and pH 9.0 for 24 h [29]. Additionally, rLAC also showed quite a good degree of stability in an acidic environment, retaining 77.1% of its original activity after incubation at pH 3.0 for 10 d. The above results demonstrate that rLAC from *B. pumilus* TCCC 11568 has a high potential for applications in a wide range of pH environments, especially under alkaline conditions.

In summary, the *B. pumilus* TCCC 11568 laccase demonstrated thermophilic features and a higher degree of thermostability than some reported *Bacillus* laccases. Therefore, this laccase has excellent potential for becoming a new industrial enzyme formulation.

### 3.4. Influence of Metal Ions and Inhibitors on the Activity of rLAC

rLAC activity was seriously affected by 5 mM Co^2+^ and Mn^2+^, retaining 24% and 9.4% of the control, respectively, but other metal ions did not lead to critical activity loss, indicating the high tolerance of rLAC towards most metal ions (Table 1). A comparable phenomenon was also found with the *B. safensis* sp. S31 laccase, which retained only 13.2% activity when incubated with 1 mM Mn^2+^ [41].

Electrolytes are required to obtain a high degree of efficiency in the industrial dyeing process. NaCl is the most extensively used electrolyte, and its concentration is approximately 25–30 g/L (around 0.5 M). Most fungal laccases are deactivated in solutions containing over 100 mM NaCl because they are intrinsically sensitive to halides [42]. This is a major barrier for applying fungal laccases to treat wastewater containing chloride ions at a high concentration. By contrast, some *Bacillus* laccases were highly tolerant to chloride, and their activities were even promoted by increased NaCl concentrations [32]. Different tolerances to halide inhibition among fungal and bacterial laccases may result from their different target localizations after protein translation. Laccases from fungal species are primarily secreted for degrading lignin, but *Bacillus* laccases anchor in the spore coat. This protein produced a high degree of stability as an indirect consequence of its evolutionary constraints, since it must adapt to a tightly packed protein coat structure [43]. Here, we found that rLAC from *B. pumilus* TCCC 11568 also exhibited a high tolerance to NaCl, and it even retained 50.7% activity of the control in a solution containing 500 mM NaCl. Similar phenomena were also reported for laccases from *B. pumilus* W3 and *B. vallismortis* fmb-103 [26,44]. Therefore, rLAC with its high salinity tolerance would demonstrate more advantages in dealing with dye effluents that are high in salinity [45]. Additionally, no vital activity loss was found during incubation with 5 mM EDTA (62.1% activity retained, Table 1). On the contrary, a complete loss was found with laccase isoforms of *Aspergillus ochraceus* and *Thermus thermophilus* in 1 mM EDTA [46,47].

The effects of some reported inhibitors, such as L-cysteine, sodium dodecyl sulfate (SDS), β-mercaptoethanol, and dithiothreitol (DTT) on their rLAC activity are also exhibited in Table 1. rLAC activity was greatly decreased by 0.5 mM DTT, L-cysteine, and β-mercaptoethanol; this phenomenon was also noted in other fungal and bacterial laccases [26,44,48]. Additionally, laccases from diverse *Bacillus* strains also demonstrated differing amounts of tolerance against the same inhibitor. SDS (0.1 mM) significantly inhibited *B. safensis* sp. S31 laccase activity, retaining only 1.3% the activity of the control [41]. On the contrary, the activity of rLAC was not even affected by SDS at a high concentration (5 mM). A similar phenomenon was also noted for the *B. vallismortis* fmb-103 laccase [26].

### 3.5. Investigation of the Thermophilic Features of rLAC via Molecular Docking and MD Simulations

**Figure 3:**
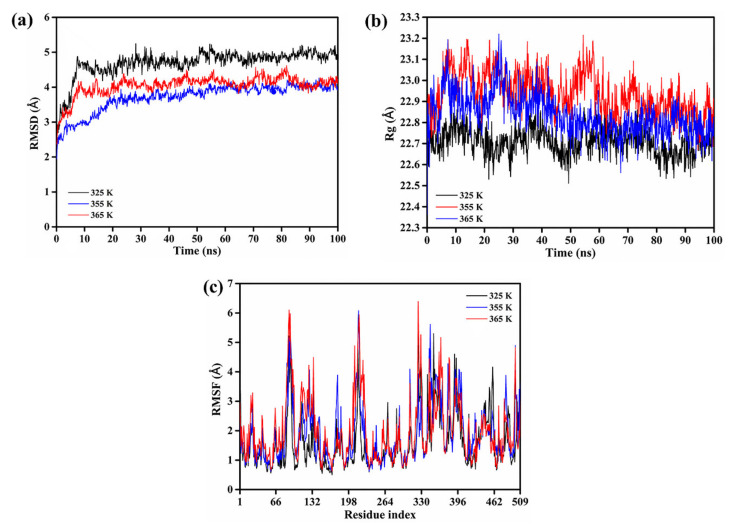
RMSD (**a**) and Rg (**b**) of rLAC binding with ABTS as a function of time (100 ns) at differing temperatures (325 K, 355 K, and 365 K); RMSF (**c**) value of each residue in rLAC-ABTS complex at differing temperatures after MD simulations.

To present a reasonable explanation for the thermo-stable features of rLAC, MD simulations were performed at 325 K, 355 K, and 365 K, respectively. The root mean square deviation (RMSD), displayed as a function of simulation time, is a key indicator for assessing the stability of protein structure upon the binding of a ligand. In general, the lower the RMSD value displayed by the protein complex during MD simulations, the higher the stability shown [49]. Our results suggested that the complex systems reached equilibrium in the 75 ns simulation (Figure 3a). The RMSD for the rLAC-ABTS complex at high temperatures (355 K and 365 K) was lower in comparison to that at a low temperature (325 K), indicating an enhanced degree of stability for the complex upon its increase in temperature.

**Figure 4:**
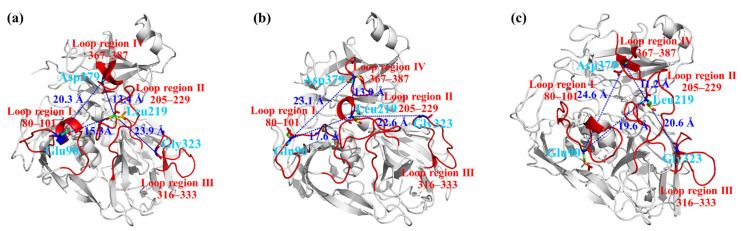
The effect of temperature on the rLAC conformation at the end of MD simulations. (**a**) 325 K; (**b**) 355 K; (**c**) 365 K. The distance among the representative amino acid residues in each loop (Glu90 in loop region I; Leu219 in loop region II; Gly323 in loop region III; Asp379 in loop region (IV) were marked with a green dashed line and specific values.

Rg, calculated as the root mean square distance from the center of mass, indicates the level of the secondary structure backbone rigidity and the compactness of the protein complex system. The Rg values of the rLAC-ABTS complex at a high temperature were larger than those at low temperature, suggesting a reduction in the compactness and structural backbone rigidity (Figure 3b). The above results suggested that the stability of the rLAC-ABTS complex is not merely determined by the protein compactness, but by a synergic conformation change. In fact, the subsequent RMSF analysis that is commonly used to characterize the structural fluctuation and protein structural integrity also displayed expansion in some of the loop regions of rLAC, indicating a decrease in protein compactness in some regions, along with an increase in temperature from 325 K to 365 K. Actually, the RMSF values for all rLAC residues under high-temperature conditions (355 K and 365 K) did not demonstrate remarkable differences to those of rLAC under mild temperature conditions (325 K), except for a few residues that were all within the loop region with the largest enhancement of RMSF values, such as Glu90, Leu219, Gly323, and Asp379 (Figure 3c and Figure 4).

**Figure 5:**
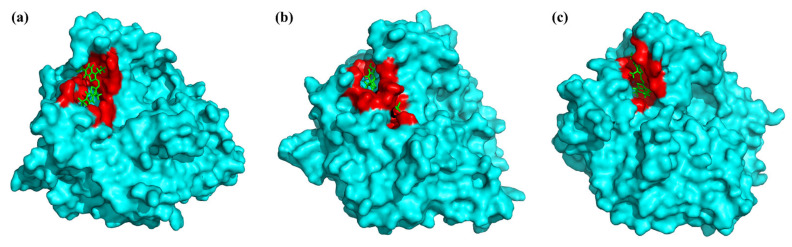
Surface representation and binding modes of the rLAC-ABTS complex. The structures were acquired from cluster analysis of the MD trajectories during 100 ns of simulation at different temperatures, (**a**) 325 K, (**b**) 355 K, and (**c**) 365 K. Surface representation of rLAC (cyan) in complex with ABTS (green) during the initial equilibrated MD trajectory. The substrate-binding pocket is presented as the red region.

The distance between residues Glu90 and Asp379, Glu90 and Leu219, Asp379, and Leu210 gradually increased along with the elevated temperature. As a result, the loop regions of I, II, and IV were far away from each other at high temperatures (Figure 4b, 355 K; Figure 4c, 365 K) at the end of the MD simulations. On the contrary, the distance between loop region II and loop region III became shorter. In conclusion, the overall conformation of rLAC did not show significant alterations at high temperatures (355 K, 365 K), but minor location adjustments in some loop regions occurred at high temperatures. As can be supposed, the good flexibility of the loop region may potentially make it simple to expose the active center, and may therefore facilitate the binding of rLAC with the substrate (ABTS) and the subsequent catalytic reaction. In fact, this speculation was also supported by observations of the surface representation and binding modes of ABTS with rLAC (Figure 5).

As can be seen from the trajectories recorded after 100 ns of MD simulations, a different spatial conformation of the binding pocket in rLAC at different temperatures was observed (Figure 5). A narrow aisle for substrate access to the active site formed under low temperatures, which obviously did not facilitate effective binding of ABTS with the rLAC in the right manner, nor subsequent catalysis (Figure 5a). By contrast, the conformation changed at high temperatures (355 K, 365 K), and a more open space was formed. As a result, ABTS could enter the binding pocket easily and it formed a tighter complex with rLAC in the active site, which was one of the most important prerequisites for an effective catalytic reaction by laccase (Figure 5b,c).

**Figure 6:**
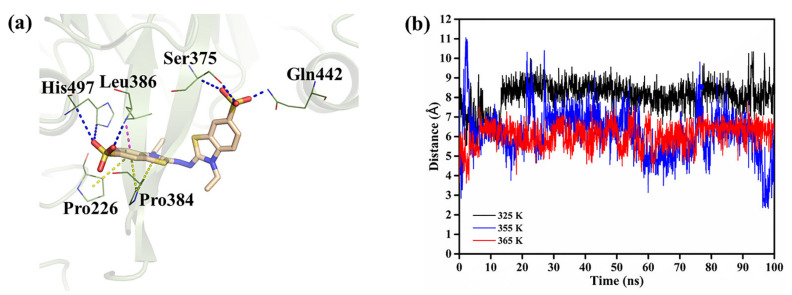
(**a**) In silico binding analysis of ABTS at the active site of rLAC and (**b**) the average distance variation between the C-atom of H497 and ABTS over 100 ns of the simulation at different temperatures.

The distance between the catalytic residue and ABTS was also analyzed to explore the influence of temperature on the catalysis performance of rLAC. The distance between the C-atom of H497 located in the active site and the C-atom of ABTS decreases along with an increase in temperature, indicating that a closer interaction between ABTS and rLAC occurred at the active center (Figure 6). In fact, the released binding free-energy analysis for rLAC-ABTS verified this phenomenon further (Table 2).

The electrostatic interaction and the van der Waals interaction made a major contribution to the production of the binding energy during the binding process of ABTS in the active center of rLAC. Moreover, the released binding energy was much higher for rLAC-ABTS at high temperatures, especially at 355 K. These results also matched the RMSD analysis in Figure 3, in which the RMSD value obtained at 355 K was the lowest, indicating a high thermostability of the rLAC-substrate complex and a much higher substrate-binding affinity at high temperature. Mollania et al. (2017) investigated the variation of the protein structure of the laccase obtained from a local *Bacillus* species (HR03) after thermal activation, using gas-phase electrophoretic mobility macromolecule analysis, far-UV CD-spectra, common biochemical methods, fluorescence analysis, etc. [50]. They speculated that the improved activity of *Bacillus* sp. HR03 laccase was not the result of oligomerization, but of the generation of a more active conformation due to the thermal treatment at a high temperature (70 °C). In other words, the incubation of *Bacillus* laccase at high temperatures did not severely affect the overall structure in an irreversible way, but instead led to a more beneficial conformation of the Cu sites and of the active site for substrate binding. According to our literature investigation, the mechanism for the high thermophilic features of *Bacillus* laccase is still not very clear. We herein presented another reasonable and vivid explanation for interpreting the thermophilic features of rLAC via docking and molecular dynamics simulations analyses. We hypothesized that the formation of a correctly matching substrate access aisle to the active site, through the minor adjustment of protein spatial conformation, was responsible for the improved catalytic activity of *Bacillus* laccase when incubated at high temperatures. As a result, the released binding energy greatly increased, due to the tighter binding of ABTS at the active site driving the accelerated catalytic reaction.

### 3.6. Dye Decolorization

Color is a vital quality attribute of food in stimulating customers’ appetites and is, therefore, one of the most important concerns for food manufacturers when considering market acceptance. Synthetic dyes as colorants are widely applied in the food industry to dye foodstuffs such as mustard, sweets, jams, cakes, and juices beverages, and they are also used in the production of drugs and cosmetics, because they have certain fascinating advantages compared to natural pigments, including brighter colors, low production costs, high water solubility, and high fading resistance against exposure to chemicals, light, and water [51].

However, large amounts of effluent with intense colors are produced after the dyeing process, as approximately 10–20% of the dyes are lost during this process [52]. Therefore, without treatment, the discharged effluents are an important source of pollution for water bodies, and bring in severe damage to aquatic ecosystems through impeding light penetration to the water bodies, which consequently reduces the photosynthetic rate of aquatic plants and the dissolved oxygen levels in the water [23,53]. In recent years, more and more bioremediation methods with favorable properties, such as higher efficiencies, lower costs, and eco-friendly properties, have been developed and applied to decolorizing dye effluents [54,55,56].

Laccases can degrade numerous environmental pollutants, including micropollutants, personal-care products, and textile dyes [6,57]. Since the typical dye effluents are generally identified via their high temperatures and high pH values (e.g., over 40 °C and around pH 9.0) [39], most fungal laccases will be deactivated under these harsh conditions; e.g., laccases from *Sclerotium rolfsii* and *T. hirsuta* can only work best with degrading indigo carmine under an acidic pH environment [45,58]. Thus, for bacterial laccases, conditions of much higher pH stability and thermostability show a higher potential for treating dye effluents.

**Figure 7:**
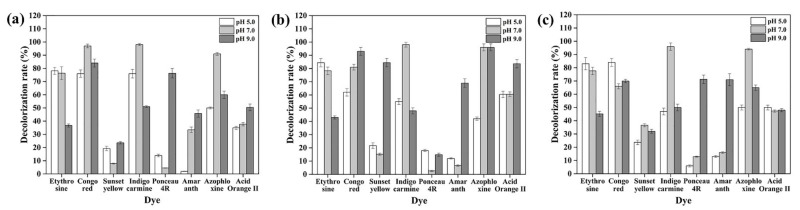
Decolorizing synthetic dyes with purified rLAC using mediator ABTS (**a**), acetosyringone (**b**), and syringaldehyde (**c**) at different pH values (5.0, 7.0, and 9.0). The operation was conducted through incubation of 80 U rLAC at 60 °C. All experiments were conducted in triplicate, and the data represent mean ± SD.

In this study, a new laccase with a high thermostability and pH stability was obtained from the newly identified bacterium *B. pumilus* TCCC 11568 (Figure 2). Therefore, we assessed its decolorization ability against several azo dyes at 60 °C and over a broad pH range, starting from pH 5.0 to 9.0 (Figure 7). The decolorization rates for each azo dye were all above 70% under optimum conditions (an appropriate redox mediator and pH environment) (Figure 7). Taking the dye Sunset Yellow for example, the decolorization rate was below 20% or 40% when using ABTS or syringaldehyde as a redox mediator, respectively. However, the decolorization rate soared up to 84.4% when incubated with syringaldehyde at pH 9.0 (Figure 7). Additionally, we also found that rLAC displayed a higher decolorizing ability against all of the dyes in a neutral (pH 7.0) or alkaline environment (pH 9.0), except for the dye etythrosine, which is more easily decolorized in an acid environment.

This differs from *B. velezensis* TCCC 111904 laccase, which was more effective for decolorizing the dyes when ABTS was used as the mediator at pH 5.5 and pH 7.0 [25]. Therefore, it was inferred that synthetic dyes with diverse structures could be efficiently decolorized by rLAC isolated from *B. pumilus* TCCC 11568 over a broad pH range (5.0–9.0) at a high temperature (60 °C) with an appropriate mediator. However, depending on the extensive specificity of the CotA laccase, no tendency of laccase could be summarized for the decolorizing activity towards synthetic dyes with different structures [59].

As is known, most dye effluents are released at high temperatures [39]. Thus, it would be helpful to decrease the unnecessary cost of cooling the dye effluents to meet the required reaction conditions due to the alkaline stability and high thermostability of rLAC. Moreover, recycling hot water after decolorization could save a lot of energy. Therefore, the *B. pumilus* TCCC 11568 laccase presented promising applications because of its excellent efficiency in decolorizing dyes under high temperatures and alkaline conditions.

## 4. Conclusions

The recombinant laccase (rLAC) of *Bacillus pumilus* TCCC 11568 demonstrated a high thermostability and pH stability over a wide pH range. The overall conformation of rLAC did not show significant alterations at a high temperature (355 K), but minor location adjustments in some loop regions occurred at a high temperature. However, those adjustments were presumed to be responsible for the formation of a more open access aisle that facilitated ABTS binding in the active site, resulting in a shorter distance from the catalytic residue and elevated binding energy. Due to its thermophilic feature, rLAC could effectively decolorize azo dyes at high temperatures over an extensive pH range in the presence of the appropriate mediator. These aforementioned features make rLAC a potential candidate for industrial applications in dye decolorization.

## Figures and Tables

**Table 1:** Effect of metal ions or inhibitors on the rLAC activity.

Metal Ions/Inhibitors	Concentration (mM)	Relative Activity (%) ^a^
None	-	100.0 ± 1.5
KCl	0.5	102.6 ± 2.7
	5	93.2 ± 1.9
CaCl_2_	0.5	96.2 ± 3.2
	5	90.8 ± 1.6
CuCl_2_	0.5	95.4 ± 2.1
	5	92.7 ± 2.0
MgCl_2_	0.5	98.0 ± 2.9
	5	91.1 ± 3.1
ZnSO_4_	0.5	97.4 ± 2.9
	5	105.5 ± 1.5
BaCl_2_	0.5	101.7 ± 1.2
	5	87.1 ± 0.5
NiSO_4_	0.5	90.9 ± 0.6
	5	105.1 ± 3.2
CoCl_2_	0.5	77.5 ± 0.5
	5	24.0 ± 0.7
FeSO_4_	0.5	75.8 ± 1.0
	5	42.1 ± 0.7
FeCl_3_	0.5	94.0 ± 3.5
	5	79.1 ± 1.8
MnCl_2_	0.5	17.5 ± 2.3
	5	9.4 ± 0.8
NaCl	0.5	105.5 ± 3.0
	5	89.5 ± 2.3
	10	79.9 ± 2.6
	100	68.7 ± 1.7
	500	50.7 ± 0.9
	1000	0
Dithiothreitol	0.5	6.2 ± 1.0
	5	0
L-Cysteine	0.5	35.2 ± 3.5
	5	27.6 ± 1.6
β-Mercaptoethanol	0.5	6.5 ± 2.0
	5	0
SDS	0.5	108.7 ± 0.7
	5	103.9 ± 2.1
EDTA	0.5	90.8 ± 1.1
	5	62.1 ± 2.8

^a^ Data represent the means ± SD (*n* = 3) relative to the untreated control samples.

**Table 2:** The released binding energy of rLAC to the ligand ABTS.

Temperature (K)	Binding Energy ^a^ (kJ/mol)	van der Waals Energy (kJ/mol)	Electrostatic Energy (kJ/mol)	Polar Solvation Energy (kJ/mol)	SASA Energy (kJ/mol)
325	−106.77 ± 21.59	−189.47 ± 20.04	−51.29 ± 20.60	155.13 ± 27.40	−21.14 ± 1.61
355	−154.14 ± 27.74	−251.44 ± 30.52	−40.50 ± 13.12	160.48 ± 17.43	−22.67 ± 1.42
365	−135.00 ± 22.68	−234.27 ± 27.52	−28.83 ± 16.34	150.44 ± 27.52	−22.34 ± 1.70

^a^ The binding energy was calculated through the summation the values of the van der Waals energy, electrostatic energy, polar solvation energy, and SASA energy.

## Data Availability

The data presented in this study are available in article and Supplementary Material.

## Supplementary Materials

The following supporting information can be downloaded at: https://www.mdpi.com/article/10.3390/foods11101387/s1, Figure S1: 16S rDNA-based phylogenetic comparison of *B. pumilus* TCCC 11568 and other *Bacillus* species. The phylogenetic tree was engineered using MEGA 6.0 software with the neighbor-joining method. The numbers at branch points suggested the percentage of bootstrap sampling from 1000 replications; Figure S2: Multiple alignments of rLAC protein sequence from *B. pumilus* TCCC 11568 and other laccases derived from the *Bacillus* genus (*B. pumilus* ATCC 7061, *B. pumilus* W3, *B. vallismortis* fmb103, *B. amyloliquefaciens* TCCC 111018, B. subtilis X1, *B. velezensis* TCCC 111904, and *B. licheniformis* ATCC 14580). The identical and similar amino acids are marked in black and solid grey, respectively. These protein sequences were obtained from NCBI database, and the alignment was carried out with DNAMAN software; Figure S3: Detection of rLAC expressed in *E. coli*. (a) Lane M: protein standard ladder; Lane 1: cell extract of *E. coli* BL21/pET-22b; Lane 2: cell extract of *E. coli* BL21/pET-lac. (b) Lane M: protein standard ladder; Lane 1: the purified rLAC. The black arrowhead indicates the rLAC band.
