# Supplementary material for: Biochemical and Structural Properties of a High-Temperature-Active Laccase from Bacillus pumilus and Its Application in the Decolorization of Food Dyes
Source: Foods. 2022 May 11;11(10):1387. doi: 10.3390/foods11101387 (PMC9141572; doi:10.3390/foods11101387)
Supplement: Supplementary file 1 [file foods-11-01387-s001.zip › foods-1695151-supplementary.pdf]

Supplementary figures

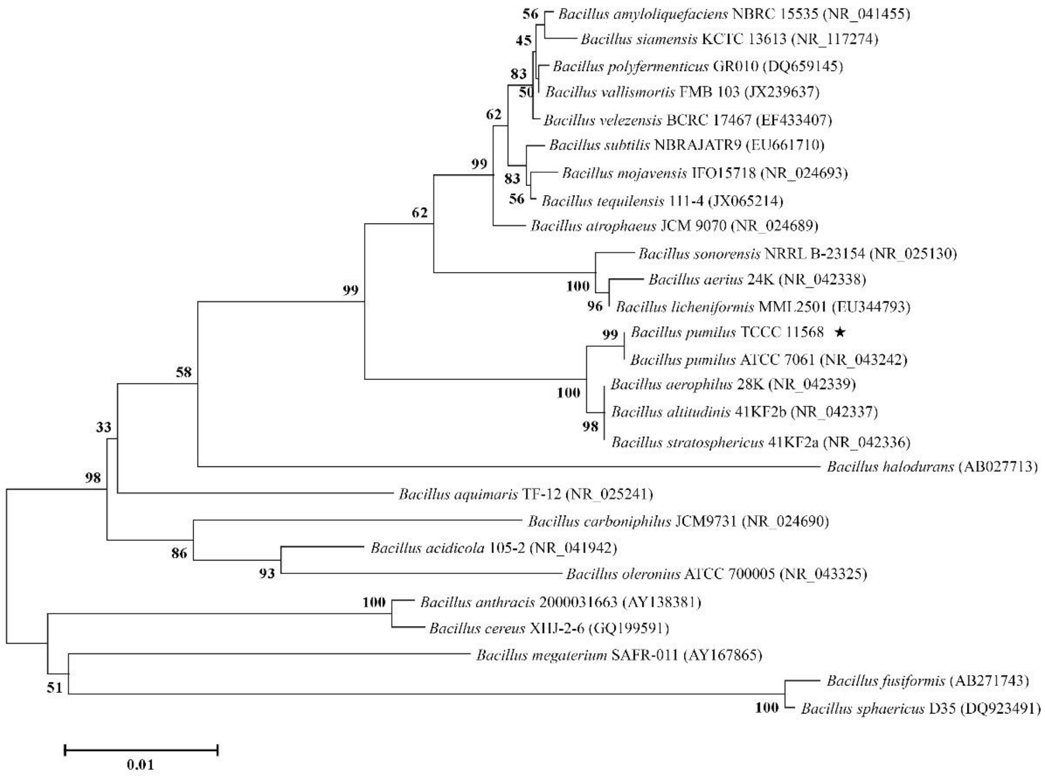

Figure S1. 16S rDNA-based phylogenetic comparison of *B. pumilus* TCCC 11568 and other *Bacillus* species. The phylogenetic tree was engineered using MEGA 6.0 software with the neighbor-joining method. The numbers at branch points suggested the percentage of bootstrap sampling from 1000 replications.

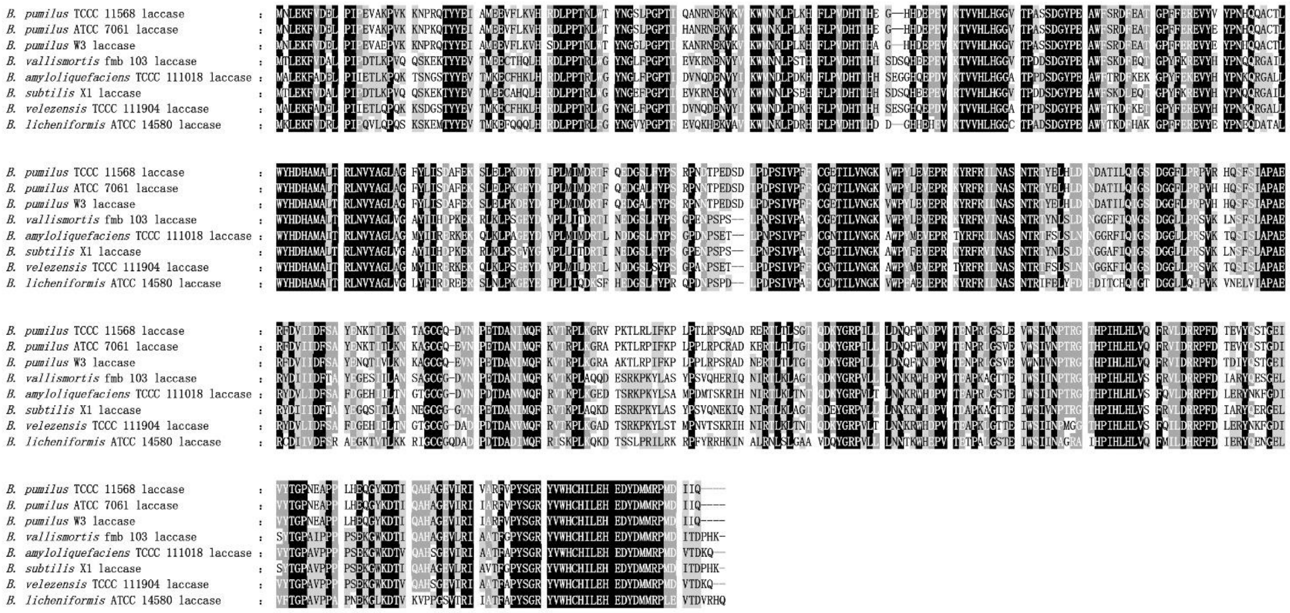

Figure S2. Multiple alignment of rLAC protein sequence from *B. pumilus* TCCC 11568 and other laccases derived from *Bacillus* genus (*B. pumilus* ATCC 7061[23], *B. pumilus* W3, *B. vallismortis* fmb103, *B. amyloliquefaciens* TCCC 111018, *B. subtilis* X1, *B. velezensis* TCCC 111904, *B. licheniformis* ATCC 14580). The identical and similar amino acids are marked in black and solid grey, respectively. These protein sequences were obtained from NCBI database, and the alignment was carried out with DNAMAN software.

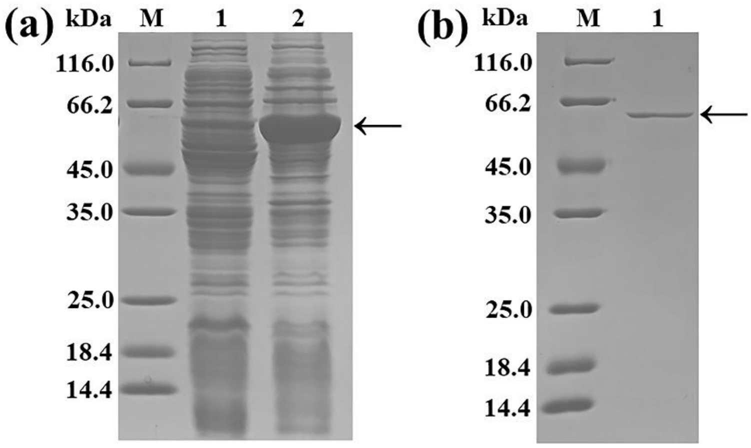

Figure S3. Detection of rLAC expressed in *E. coli*. (a) Lane M: protein standard ladder; Lane 1: cell extract of *E. coli* BL21/pET-22b; Lane 2: cell extract of *E. coli* BL21/pET-lac. (b) Lane M: protein standard ladder; Lane 1: the purified rLAC. Black arrowhead indicates the rLAC band.
